# Phytochemical composition and antimicrobial activities of the essential oils and organic extracts from *pelargonium graveolens* growing in Tunisia

**DOI:** 10.1186/1476-511X-11-167

**Published:** 2012-12-05

**Authors:** Anis Ben Hsouna, Naceur Hamdi

**Affiliations:** 1 College of Science and Arts, Al-Rass, Qassim University, P.O. Box 53, Qassim, Saudi Arabia; 2 Bio-pesticides Team (LPIP), Center of Biotechnology of Sfax, P.B. 1177, Sfax, 3018, Tunisia; 3 Higher Institute of Environmental Science and Technology, Borj-Cédria, TUNISIA" & "University of Carthage, Carthage, Tunisia

**Keywords:** *P. graveolens*, Antimicrobial activity, Essential oils, Organic extracts, GC/MS

## Abstract

**Background:**

*Pelargonium graveolens* (*P. graveolens*) L. is an aromatic and medicinal plant belonging to the *geraniacea* family.

**Results:**

The chemical compositions of the essential oil as well as the *in vitro* antimicrobial activities were investigated. The GC-MS analysis of the essential oil revealed 42 compounds. Linallol L, Citronellol, Geraniol, 6-Octen-1-ol, 3,7-dimethyl, formate and Selinene were identified as the major components. The tested oil and organic extracts exhibited a promising antimicrobial effect against a panel of microorganisms with diameter inhibition zones ranging from 12 to 34 mm and MIC_s_ values from 0.039 to10 mg/ml. The investigation of the phenolic content showed that EtOAc, MeOH and water extracts had the highest phenolic contents.

**Conclusion:**

Overall, results presented here suggest that the essential oil and organic extracts of *P. graveolens* possesses antimicrobial and properties, and is therefore a potential source of active ingredients for food and pharmaceutical industry.

## Background

In recent years, there has been considerable interest in the search for antioxidants and antimicrobial compounds from natural sources to control human and plant diseases [1]. Natural antioxidants inhibit the oxidative damage of food products and may prevent inflammatory conditions [2], ageing and neurodegenerative disease [3]. The market constantly addresses its attention to secondary metabolites produced by plants to check their properties and to evaluate their possible use in the industry. Also, scientific interest in these metabolites is increasing today with the search of new antimicrobial agents due to the increasing development of the resistance pattern of microorganisms to most currently used antimicrobial drugs. A number of natural products such as phenolics, flavonoids, coumarins, curcuminoids or terpenes have been characterised as metabolites which allow easy transport across cell membranes to induce different biological activities, including antioxidant, anti-inflammatory and anticholinesterase effects [4].

*Pelargonium graveolens* (*P. graveolens*) is a plant about which technical and scientific knowledge is scarce. It belongs to the *Geraniaceae* family, and its leaves are popularly used as flavouring, insect repellent, in perfume and in aromatherapy for the treatment of gastrointestinal diseases, throat infections, and bleeding [5]. Previous reports have documented the antimicrobial activity of some *Geraniaceae* extracts and their constituents against several bacterial and fungal pathogens [6]. These studies have provided little information on the identity of the chemical constituents of the essential oil of this species or its biological activities.

The aim of the present study was to investigate the chemical composition of the Tunisian *P. graveolens* essential oil and its potent antimicrobial effect as well as that of the organic extracts. Total phenolic and flavonoids contents of the extracts were also determined.

## Materials and methods

### Chemicals

Gentamicin and Amphotericin B were purchased from Sigma-Chemical (France). Folin-Ciocalteu reagent, sodium carbonate (Na_2_CO_3_) and other solvents were of analytical grade and were freshly prepared in distilled water.

### Plant material

*P. graveolens* leaves were collected from Chebba (Tunisia, 35.23° N and 11.11° E) in April 2009. After the botanical identification of the species, a voucher specimen (LBPes 08) was deposited in the Herbarium of the Laboratory of Biopesticides (Center of Biotechnology of Sfax, Tunisia) for future reference.

### Preparation of plant organic extracts

A portion of dried leaves (100 g) of *P. graveolens* was placed in a soxhlet apparatus. Extraction was performed with 500 ml of an appropriate solvent with increased polarity for 48 h at a temperature not exceeding the boiling point of the solvent. The extract was filtered through a 45 *μ*m filter and concentrated under vacuum. In this experiment three solvents were used: *n*-hexane, ethyl acetate and methanol [7]. The resulting three solutions were concentrated *in vacuous* to dryness to give *n*-hexane extract HE (4 g), ethyl acetate extract EtOAcE (10 g) and methanol extract MeOHE (12 g). The remaining residue was extracted by water infusion and lyophilised to give water extract WE (15 g) and the resulting powder material was stored until tested. The stock solutions were kept at 4°C in the dark until further analysis.

### Essential oil extraction

The oil extraction was obtained from 0.5 kg fresh plant by steam distillation during 3 h using a Clevenger-type apparatus [7,8]. The aqueous phase was extracted with dichloromethane (3 × 50 ml) and dried with anhydrous sodium sulphate. For the determination of the procedure yield, the solvent was evaporated using a rotavapory vacuum evaporator to afford 2.5 g. The resulting essential oil (EO) was stored at 4°C prior to further analyses. The EO was solubilised in *n*-Hexane for gas chromatography and mass spectrometry analysis.

### Total phenolic content

Total phenolic content (TPC) was determined using the Folin-Ciocalteu method [9] adapted to a microscale. Briefly, 10 μl of diluted sample solution were shaken for 5 min with 50 μl of Folin-Ciocalteu reagent. Then 150 μl of 20% Na_2_CO_3_ were added and the mixture was shaken once again for 1 min. Finally, the solution was brought up to 790 μl by the addition of distilled water. After 90 min, the absorbance at 760 nm was evaluated using a spectrophotometer SmartSpecT^m^3000 (Bio-Rad; Hercules, CA, USA). Gallic acid was used as an internal standard for the calibration curve. The phenolic content was expressed as mg of gallic acid equivalent per gram of dry sample (mg GAE/g) using the linear equation based on the calibration curve.

### Determination of total flavonoids content

The flavonoids content in the extracts was determined spectrophotometrically according to Quettier-Deleu et al. [10], using a method based on the formation of a complex flavonoid-aluminium, having the maximum absorption at 430 nm. The flavonoids content was expressed in mg of quercetin equivalent per gram of dry plant extract (mg QE/g).

### Gas chromatography–mass spectrometry (GC–MS)

The EO analysis was performed on a GC–MS HP model 6980 inert MSD (Agilent Technologies, J&W Scientific Products, Palo Alto, CA, USA), equipped with an Agilent Technologies capillary HP-5MS column (60 m length; 0.25 mm i.d.; 0.25 mm film thickness), and coupled to a mass selective detector (MSD5973, ionization voltage 70 eV; all Agilent, Santa Clara, CA) [11]. The carrier gas was He and was used at 1.2 mL/min flow rate. The oven temperature program was as follows: 1 min at 100°C ramped from 100 to 280°C at 5°C min^_1^ and 25 min at 280°C. The chromatograph was equipped with a split/split less injector used in the split less mode. Components identification was assigned by matching their mass spectra with Wiley 275 L and NIST library data, standards of the main components [12].

## Antimicrobial activity

### Microorganisms and growth conditions

Authentic pure cultures of bacteria and fungi were obtained from international culture collections (ATCC) and the local culture collection of the Center of Biotechnology of Sfax, Tunisia. They included Gram-positive bacteria: *Bacillus subtilis* ATCC *6633, Bacillus cereus* ATCC 14579*, Staphylococcus aureus* ATCC 25923*, Staphylococcus epidermidis* ATCC 12228*, Enterococcus faecalis* ATCC 29212*, Micrococcus luteus* ATCC 1880*, Listeria monocytogenes (*food isolate 2132*)* and Gram-negative bacteria: *Salmonella enterica* (food isolate)*, Klebsiella pneumoniae* ATCC 10031*.*

The following *fungal strains* were also tested: *Aspergillus niger* CTM 10099, *Aspergillus flavus* (food isolate), *Fusarium graminearum* (ISPAVE 271), *Fusarium oxysporum* (CTM10402), *Fusarium culmorum* (ISPAVE 21w), *Rhizopus nigricans* (CTM10150), and *Alternaria alternata* (CTM 10230).

The bacterial strains were cultivated in Muller-Hinton agar (MH) (Oxoid Ltd, UK) at the appropriate temperature for each strain at 37°C and fungi were cultured on Potatoes Dextrose agar (PDA) medium at 28°C. Working cultures were prepared by inoculating a loopful of each test bacteria in 3 ml of Muller-Hinton broth (MH) (Oxoid Ltd, UK) and were incubated at 37°C for 12 hours. For the test, final inoculum concentrations of 10^6^ CFU/ml bacteria were used. Fungal spore suspensions were collected from the surface of such fungal colonies by gentle scraping with a loop and suspended in 10 ml Potato Dextrose broth (PDB).

This suspension was mixed vigorously by vortexing for 15–20 min. The spore suspension stock was diluted to obtain a concentration of 10^6^ spores/ml (measured by Malassez blade).

### Agar diffusion method

The antimicrobial activity of the *P. graveolens* was evaluated by means of agar-well diffusion assay according to Güven et al. [13] with some modifications. Fifteen millilitres of the molten agar (45°C) were poured into sterile petri dishes (Ø 90 mm). Working cell suspensions were prepared and 100 μl were evenly spreaded onto the surface of the agar plates of Mueller-Hinton agar (Oxoid Ltd, UK) for bacteria, or potatoes dextrose agar medium (Oxoid Ltd, UK) for fungi. Once the plates had been aseptically dried, 06 mm wells were punched into the agar with a sterile Pasteur pipette. The *P. graveolens oils* and extracts were dissolved in dimethylsulfoxide/water (1/1) and sterile water to a final concentration of 50 mg/ml. Thus, 50 μl were placed into the wells and the plates were incubated at 37°C for 24 h for bacterial strains and 72 h for fungi at 28°C. Gentamicin (15 μg/wells), Amphotericin B (20 μg/wells) and DMSO served as positive and negative control. Antimicrobial activity was evaluated by measuring the diameter of circular inhibition zones around the well. Tests were performed in triplicate.

### Determination of MIC and MFC

Minimum inhibitory concentrations (MICs) of *P. graveolens* were determined according to Gulluce et al. [14] against a panel of 21 microorganisms representing different species of different ecosystems. The test was performed in sterile 96-well microplates with a final volume in each microplate well of 100 μl. A stock solution of the *P. graveolens* (50 mg/ml) was prepared in dimethylsulfoxide/water (1/9). The inhibitory activity of the EO and organic extracts was properly prepared and transferred to each well in order to obtain a twofold serial dilution of the original sample and to produce the concentration range of 0.039-10 mg/ml. To each test well 10 μl of cell suspension were added to final inoculum concentrations of 10^6^ CFU/ml for bacteria and 10^5^ spores/ml for fungus. Positive growth control wells consisted of bacteria or fungi only in their adequate medium. Dimethylsulfoxide/water (1/9) was used as negative control. The plates were then covered with the sterile plate covers and incubated at 37°C for 24 h for bacterial strains and 72 h for fungi at 28°C. The MIC was defined as the lowest concentration of the total essential oil at which the microorganism does not demonstrate visible growth after incubation. As an indicator of microorganism growth, 25 μl of Thiazolyl Blue Tetrazolium Bromide (MTT), indicator solution (0.5 mg/ml) dissolved in sterile water were added to the wells and incubated at 37°C for 30 min [15]. The colourless tetrazolium salt acts as an electron acceptor and is reduced to a red-coloured formazan product by biologically active organisms. Where microbial growth was inhibited, the solution in the well remained clear after incubation with MTT. The minimum fungicidal concentrations (MFCs) were determined by serial subcultivation of 10 μl in Potatoes Dextrose agar (PDA) plates and incubated for 72 h at 28°C. The lowest concentration with no visible growth was defined as the MFC, indicating ≥ 99.5% killing of the original inoculum. DMSO and ethanol were used as a negative control.

The determinations of MIC, MBC and MFC values were done in triplicate.

### Statistical analysis

The experimental results concerning this study were expressed as means ± standard deviation of three parallel measurements. The significance of difference was calculated by Student’s *t* test, and values *p* < 0.05 and *p* < 0.001 were considered to be significant and highly significant respectively. Correlation and regression analysis was carried out using EXCELL program (Microsoft Corporation, USA).

## Results and discussion

### Chemical composition of the leaf essential oil

The hydrodistillation of *P. graveolens* leaves gave yellow to brownish oil. GC–MS analyses of the essential oil led to the identification of 42 different compounds, listed in Table 1 according to their elution order on a HP-5MS column. The oil contained a complex mixture mainly of monoterpene hydrocarbons with some other essential phytochemicals. The prominent components were Linalool (6.54%), Citronellol (27.53%), Geraniol (25.85%), 6-Octen-1-ol, 3,7-dimethyl-, formate (8.75) and **Δ**Selinene (8.15%).

**Table 1:** **Chemical composition of essential oil isolated by hydrodistillation from *P. graveolens *leaves**

**Pic**	**Compounds**^**a**^	**Rt (min)**	**% of abundance**
1	α-Pinene	8.829	0.28
2	Δ.3-Carene	11.724	0.07
3	E-β-Ocimene	12.033	0.17
4	Linalool	14.030	6.54
5	E-ROSE OXIDE	14.264	0.32
6	Z-ROSE OXIDE	14.739	0.12
7	Isomenthone	15.523	0.18
8	L-menthone	15.912	6.22
9	Menthomenthol	16.399	0.17
10	α-terpineol	16.639	0.30
11	Citronellol	17.961	27.53
12	Z-Citral	18.098	0.55
13	Geraniol	18.779	25.85
14	6-Octen-1-ol, 3,7-dimethyl-, formate	19.111	8.75
15	2,6-Octadien-1-ol, 3,7-dimethyl-, formate	19.780	2.70
16	Z-2,6-Dimethyl-2,6-octadiene	21.062	0.21
17	α.-Copaene	21.714	0.18
18	acetate de Geranyl	21.869	0.74
19	β. BOURBONENE	21.961	0.22
20	E-(.β.)-CARYOPHYLLENE	22.853	0.33
21	Α.-Cubebene	23.580	0.11
22	Α-Humulene	23.711	0.12
23	Aromadendrene	23.895	0.17
24	Acetate	24.146	0.57
25	Δ-germacrene	24.415	1.50
26	Ledene	24.747	0.70
27	Acide Propanoique	25.090	0.15
28	Δ-Cadinene	25.405	0.59
29	Α-agarofuran	25.989	0.19
30	Acide Butanoique	26.206	0.18
31	Acide-2-Butenoique	26.887	1.43
33	geranyl propionate	27.213	0.15
34	Ledol	27.339	0.15
35	Δ-Selinene	27.803	8.15
36	cadina-1,4-diene	27.888	0.25
37	γ-Eudesmol	27.974	0.12
38	Valencene	28.072	0.32
39	α-Cadinol	28.209	0.39
40	tau.-Cadinol	28.461	1.21
41	Z-2,6-Dimethyl-2,6-octadiene	28.621	0.19
42	tiglate de geranyl	29.433	1.92

^a^ Identification of components based on GC-MS Wiely 7.0 version library.

^b^ Rt: retention time.

### Extraction yield, total phenolic and flavonoids contents determination

The extraction yield varied between 5.7% and 21.5% depending on the extraction solvent with the following order: WE > MeOHE > EtoAcE > HE (Table 2). Naturally occurring phenolic compounds, including phenolic acids, flavonoids, tannins, coumarins and others have been reported to be significantly associated with the biological activities of plant and food extract [16]. In the present study, the total phenolic compounds in the organic extracts of *P. graveolens* were determined. These compounds showed differences in their total contents depending on solvents polarities. The TPC values of the successive *P. graveolens* leaves extracts varied between 140.75 and 145 mg GAG/g and were in the following order: MeOHE > EtoAcE > WE > HE (Table 2). Total flavonoids content ranged from 10.90 to 21.75 mg QE/g. Water extract had the highest level with 21.75 QE/g, while hexane had the lowest amounts (10.90 mg QE /g). As can be seen, high phenol content was not always accompanied by high flavonoid concentrations. This result was in agreement with the reports of Boukhris et al.; Hertog et al. and Yen et al., which proved that the methanol is the most suitable solvent for the extraction of phenolic compounds [17,19]. The total phenolic content level in our case may be related to the hard climate conditions (high temperature and solar exposure, dryness, short growing season). Based on the phenolic and flavonoid content, methanol was the best solvents to be used for *P. graveolens* phenolic compounds extraction compared to the other solvents due to their suitable polarities. These results revealed that *P. graveolens* extracts contains more significant phenolics and flavonoids contents than those of some plants, commonly used as antioxidants in previous studies [20,21].

**Table 2:** **Yields extracts, amounts of total flavonoids, and total phenolic compounds of *P. graveolens *leaves crude extracts**

**Extract**	**Yield (%)**	**Phenolic content mg GAE/g**	**Flavonoid content mg QE/g**
*n-*Hexane	5.70	59.50 ± 3.95	11.50 ± 1.95
Ethyl acetate	14.25	144.25 ± 2.75	12.25 ± 3.70
Methanol	17.25	145 ± 4.00	10.90 ± 3.00
Water	21.50	140.75 ± 8.25	21.75 ± 4.70

(mg GAE /g): mg of gallic acid equivalent per g of dry plant extract.

(mg QE/g): mg of quercetin equivalent per g of dry plant extract.

### Antibacterial activity

Plant extracts are widely claimed to have a broad-spectrum antibacterial activity and are considered as a main source for the search of lead compounds. The antimicrobial activities of *P. graveolens* essential oil and extracts against the tested microorganisms were qualitatively and quantitatively assessed by the presence or absence of inhibition zones, MIC and MBC values. The results are given in Tables 3 and 4. Among the tested extracts, only EtOAcE and MeOHE extracts exhibited an antimicrobial activity. HE and WE remained inactive in the range of the used concentration (4 mg/wells). Active extracts showed a potent antimicrobial activity against both Gram-positive and Gram-negative bacteria. The inhibition zone diameters and MIC values were in the range of 17–27 mm and 0.039-1.25 mg/ml for the ethyl acetate extract and 12–20 mm and 0.078-2.5 mg/ml for the methanol one, respectively. Compared to the antibacterial activity of MeOH extract, and of the EtOAc showed a significant one (p < 0.05). Negative control did not show any inhibitory effect against the tested bacteria.

**Table 3:** **Antimicrobial activities of *P. graveolens *organic extracts and essential oil against fungi, foodborne and spoiling bacteria (4 mg /well)**

	**Inhibition zones diameters (mm)**^**a**^					
**Extracts**	**HE**	**EtOAc**	**MeOH**	**WE**	**EO**	**Genta**^**b**^
**Strains**						
***Bacterial strains***						
**Gram positive**						
*Bacillus subtilis* ATCC 6633	-	27 ± 0.1	14 ± 0.4	-	22 ± 0.0	20 ± 0.2
*Bacillus cereus* ATCC 14579	-	17 ± 0.3	12 ± 0.8	-	26 ± 0.2	20 ± 0.4
*Staphylococcus aureus* ATCC 25923	-	25 ± 0.0	13 ± 0.6	-	24 ± 0.3	25 ± 0.8
*Staphylococcus epidermis* ATCC 12228	-	24 ± 0.6	20 ± 0.3	-	14 ± 0.4	20 ± 0.5
*Enterococcus faecalis* ATCC29212	-	18 ± 0.1	0	-	20 ± 0.3	12 ± 0.2
*Micrococcus luteus* ATCC 1880	-	21 ± 0.4	18 ± 0.5	-	15 ± 0.6	20 ± 0.7
*Listeria monocytogenes* (food isolate 2132)	-	19 ± 0.2	18 ± 0.1	-	0	15 ± 0.0
**Gram negative**						
*Salmonella enterica* (food isolate)	-	24 ± 0.2	14 ± 0.3	-	14 ± 0.1	18 ± 0.8
*Klebsiella pneumoniae* ATCC 10031	-	18 ± 0.2	15 ± 0.1	-	13 ± 0.3	12 ± 0.5
***Fungal strains***						
*Aspergillus niger* CTM 10099	-	34 ± 0.1	16 ± 0.2	-	18 ± 0.7	15 ± 0.9
*Aspergillus flavus* (food isolate)	-	24 ± 0.2	18 ± 0.5	-	22 ± 0.2	10 ± 0.3
*Fusarium graminearum* (ISPAVE271)	-	15 ± 0.7	13 ± 0.4	-	16 ± 0.4	14 ± 0.5
*Fusarium oxysporum* (CTM 10402)	-	14 ± 0.3	23 ± 0.7	-	26 ± 0.1	14 ± 0.2
*Fusarium culmorum* (ISAVE21w)	-	14 ± 0.2	17 ± 0.5	-	18 ± 0.6	12 ± 0.7
*Alternaria alternata* (CTM 10230)	-	14 ± 0.1	17 ± 0.6	-	17 ± 0.5	14 ± 0.6
*Rhizopus nigricans* (CTM10150)	-	17 ± 0.5	22 ± 0.2	-	26 ± 0.1	Nt

Values are given as mean ± S.D. of triplicate experiment.

^a^ Diameters of inhibition zone of various *P. graveolens* organic and essential oil extracts including 6 mm disk diameters.

**Abreviations:** Genta^b^: Gentamicin was used as a standard antibiotic at a concentration of 15 μg/well.

Ampho^c^: Amphotrycine B was used as a standard antifungal at a concentration of 20 μg/well.

**Table 4:** **Determined MIC, MBC and MFC values of *P. graveolens *organic extracts and essential oil against bacterial species and fungal strains**

**Bacteria**	**Extracts**	**EtOAcE**		**MeOHE**		**EO**	
**Gram positive**		**MIC**	**MBC**	**MIC**	**MBC**	**MIC**	**MBC**
*Bacillus subtilis* ATCC 6633		0.156	5	0.078	0.156	5	5
*Bacillus cereus* ATCC 14579		0.039	20	0.156	20	10	20
*Staphylococcus aureus* ATCC 25923		0.625	20	2.5	10	0.312	0.625
*Staphylococcus epidermis* ATCC 12228		0.156	0.312			5	10
*Enterococcus faecalis* ATCC29212		1.25	10	0	0	2.5	20
*Micrococcus luteus* ATCC1880		0.312	20	2.5	10	10	10
*Listeria monocytogenes* (food isolate 2132)		0.156	0.625	2.5	5	2.5	10
**Gram negative**							
*Salmonella enterica* (food isolate)		0.078	2.5	0.312	5	5	5
*Klebsiella pneumoniae* ATCC 10031		0.312	5	0.312	10	10	10
***Fungal strains***		**MIC**	**MFC**	**MIC**	**MFC**	**MIC**	**MFC**
*Aspergillus niger* CTM 10099		0.312	2.5	1.25	20	0.625	5
*Aspergillus flavus* (food isolate)		0.625	5	2.5	20	1.25	10
*Fusarium graminearum* (ISPAVE271)		1.25	10	0.625	5	0.312	5
*Fusarium oxysposium* (ISAVE21w)		1.25	5	0.312	2.5	0.156	5
*Alternaria alternata* (CTM 10230)		0.625	5	1.25	10	0.625	5
*Rhizopus nigrcans* (CTM 101052)		1.25	20	1.25	20	0.	10

Values are expressed on mg/ml.

MIC: Minimum inhibitory concentrations.

MBC: Minimum bactericidal concentrations.

MFC: Minimum fungicidal concentrations.

EtOAcE: ethyl acetate extract.

MeOHE: methanol extract.

EO: essential oil.

Also, our results showed that the essential oil of *P. graveolens* had great potential for antimicrobial activity against a panel of microorganisms. The maximum inhibition zone diameters and MIC values for bacterial strains, which were sensitive to the EO, were in the range of 13–26 mm and 0.312–10 mg/ml, respectively (Tables 3 and 4).

On the basis of inhibition zone diameters, MIC and MBC values, *Staphylococcus aureus* was more sensitive to the EO than the other Gram positive bacteria with inhibition zone diameter of 24 mm and MIC and MBC values of 0.312 and 0.625 mg/ml respectively. Our results are in good agreement with the findings of Hussain [22] who reported that Gram positive bacteria are more sensitive to plant essential oils than Gram negative bacteria, especially *E. coli*[23]. The resistance of Gram negative bacteria against essential oils has been attributed to the presence of a hydrophilic outer membrane containing a hydrophilic polysaccharide chain, which acts as a barrier hydrophobic essential oil [24]. The intense antimicrobial properties of essential oils from the leaves of *P. graveolens* was suspected to be associated with their high contents of oxygenated monoterpene appeared more active against the tested Gram positive than Gram negative bacteria. This result was in agreement with many studies realized on other plant species like *E. robusta*, *E. alba*, *E. camadulensis, E. citriodora, E. globulus, E. saligna*[23].

Furthermore, EtOAc and MeOHE of *P. graveolens* showed a potent inhibition for *B. subtilis, L. monocytogenes* and *S. enterica* with MIC of 0.156 and 0.078 mg/ml respectively. Infections caused by these bacteria, especially those with multi-drugs resistance, are among the most difficult to treat with conventional antibiotics. In the current study, the growth of *B. subtilis* was remarkably inhibited by the ethyl acetate extract of *P. graveolens* (IZ = 27 mm). These results show that *P. graveolens* EtOAc organic extract can be used to minimize problems of drug resistance and protect foods against multiple pathogenic bacteria. Our data suggest that antibacterial activities might be related to the phenolic compounds found in the EtOAC and MeOH extracts.

### Antifungal activity

Contamination by *Aspergillus*, *Fusarium* and *Alternaria* species especially with their respective mycotoxins is considered as a challenge for the pharmaceutical and food industries. The current study reports the capacity of the crude extracts and essential oil of *P. graveolens* to control *Aspergillus sp*., *Fusarium sp.* and *Alternaria alternata* strains. Among the tested extracts, only MeOH and EtOAc exhibited an antifungal activity. Results showed a strong inhibitory effect of EtOAc on the growth of *A. niger* and *A. flavus* with inhibition zone diameters of 24–34 mm and MIC values of 0.312 and 0.625 mg/ml respectively (Tables 3 and 4). Also, the *P. graveolens* EO exhibited an antifungal activity against *Fusarium* and *Aspergillus* sp. such as *A. niger* and *A. flavus* which are responsible for food spoilage. The maximum inhibition zone diameters were 14 – 34 mm and MIC values ranged from 0.078 to 1.25 mg/ml (Table 3 and Table 4). The inhibition zone of the EO against *Aspergillus* sp. was recorded as 18–22 mm and the minimum inhibition concentration values were 0.625-1.25 mg/ml. Essential oil is a complex mixture of compounds with low molecular weights and the antimicrobial effect of total oil is related to one or a few principles

To the best of our knowledge, this is the first study providing data on the antifungal activity of the essential oil and organic extracts of *P. graveolens* plants evaluated against a wide range of fungal strains. Findings revealed that the extracts of this aromatic plant could be useful as an alternative antimicrobial agent in natural medicine for the treatment of many infectious diseases.

## Conclusion

*P. graveolens* is a medicinal and edible herb growing wild in Tunisia and used for a long time in Mediterranean cuisine, not only to improve or modify food flavor, but also to avoid its deterioration. In the present work we report the chemical composition of the Tunisian *P. graveolens* essential oil which revealed 42 main components. We also investigated the antimicrobial activities of the essential oil and organic extracts from this herb. The ethyl acetate extract of *P. graveolens*, revealed a very important *in vitro* antibacterial activity on the studied bacteria, confirmed by low minimum inhibitory concentrations (MIC). It can therefore be used as a natural antimicrobial agent for the treatment of several infectious diseases. Furthermore studies are needed for the purification of the methanol extract and the identification of active molecules.

Our results are a contribution to a better valorisation of this medicinal plant. Several other biological tests will be worthwhile to search for more eventual activities of this plant to characterize active principles, and assess toxicity by laboratory assays.

## Competing interests

The authors declare that they have no competing interests.

## Authors’ contributions

ABH and NH designed the experiments, analyzed the data and drafted the manuscript. All authors read, correct and approve the final manuscript.

## References

[B1] Tepe B Daferera D Sokmen A Sokmen M Polissiou M Antimicrobial and antioxidant activities of essential oil and various extracts of salvia tomentosa miller (lamiaceae) Food Chem 2005 90 333 340 10.1016/j.foodchem.2003.09.013

[B2] Khanna D Sethi G Ahn KS Pandey MK Kunnumakkara AB Sung B Aggarwal A Aggarwal BB Natural products as a gold mine for arthritis treatment Curr Opin Pharmacol 2007 7 344 351 10.1016/j.coph.2007.03.002 17475558

[B3] Fusco D Colloca G Lo Monaco MR Cesari M Rationale for antioxidant supplementation in sarcopenia J Clin Interv Aging 2007 2 377 387 PMC2685276 18044188

[B4] Loizzo MR Tundis R Conforti F Saab AM Statti GA Menichini F Composition and α-amylase inhibitory effect of essential oils from cedrus libani Fitoterapia 2007 78 323 326 10.1016/j.fitote.2007.03.006 17499940

[B5] Saraswathi J Venkatesh K Baburao N Hilal MH Roja Rani A Phytopharmacological importance of pelargonium species Journal of Medicinal Plants Research 2011 5 2587 2598

[B6] Mativandlela SPN Lall N Meyer JJN Antibacterial, antifungal and antitubercular activity of (the roots of) Pelargonium reniforme (CURT) and Pelargonium sidoides (DC) (Geraniaceae) root extracts South African Journal of Botany 2006 72 232 237 10.1016/j.sajb.2005.08.002

[B7] Ben Hsouna A Trigui M Ben Mansour R Mezghani Jarraya R Damak M Jaoua S Chemical composition, cytotoxicity effect and antimicrobial activity of ceratonia siliqua essential oil with preservative effects against Listeria inoculated in minced beef meat Int J Food Microbiol 2011 148 66 72 10.1016/j.ijfoodmicro.2011.04.028 21601302

[B8] Sartoratto A Machado ALM Delarmelina C Figueira GM Duarte MCT Rehder VLG Composition and antimicrobial activity of essential oils from aromatic plants used in brazil Braz J Microbiol 2004 35 275 280 10.1590/S1517-83822004000300001

[B9] Ben Hsouna A Culioli MG Blache Y Jaoua S Antioxidant constituents from lawsonia inermis leaves: isolation, structure elucidation and antioxidative capacity Food Chem 2011 125 193 200 10.1016/j.foodchem.2010.08.060

[B10] Quettier-Deleu C Gressier B Vasseur J Dine T Brunet C Luyck M Cazin M Cazin JC Bailleul F Trotin F Phenolic compounds and antioxidant activities of buckwheat (fagopyrum esculentum moench) hulls and flour J Ethnopharmacol 2000 72 35 42 10.1016/S0378-8741(00)00196-3 10967451

[B11] Fendri I Chaari A Dhouib A Jlassi B Carriere F Sayadi S Abdelkafi S Isolation, identification and characterization of a new lipolytic pseudomonas sp., strain AHD-1, from Tunisian soil Environ Technol 2010 31 87 95 10.1080/09593330903369994 20232682

[B12] Barouh N Abdelkafi S Fouquet B Pina M Scheirlinckx F Carrière F Villeneuve P Neutral lipid characterization of non-water-soluble fractions of carica papaya latex Journal of American Oil Chemist Society 2010 87 987 995 10.1007/s11746-010-1582-1

[B13] Güven K Yücel E Çetintaş F Antimicrobial activities of fruits of crataegus and pyrus species Pharm Biol 2006 44 79 83 10.1080/13880200600591253

[B14] Gulluce M Sahin F Sokmen M Ozer H Daferera D Sokmen A Polissiou M Adiguzel A Ozkan H Antimicrobial and antioxidant properties of the essential oils and methanol extract from mentha longifolia L. ssp. Longifolia Food Chem 2007 103 1449 1456 10.1016/j.foodchem.2006.10.061

[B15] Shojaee-Aliabadi S Hosseini H Mohammadifar MA Mohammadi A Ghasemlou M Mahdi Ojagh S Marzieh Hosseini S Khaksar R Characterization of antioxidant-antimicrobial κ-Carrageenan films containing satureja hortensis essential oil Int J Biol Macromol 2012 In Press 10.1016/j.ijbiomac.2012.08.026 22959956

[B16] Gupta S Prakash J Studies on indian green leafy vegetables for their antioxidant activity Plant Foods Hum Nutr 2009 64 39 45 10.1007/s11130-008-0096-6 18985454

[B17] Boukhris M Simmonds MSJ Sayadi S Bouaziz M Chemical composition and biological activities of polar extracts and essential oil of rose-scented geranium, Pelargonium graveolens Phytother Res 2012 10.1002/ptr.4853 23027699

[B18] Hertog MGL Hollman PCH Van de Putte B Content of potentially anticarcinogenic flavonoids of tea infusions, wines, and fruit juices Journal Agricultrual Food Chemistry 1993 41 1242 1246 10.1021/jf00032a015

[B19] Yen G Wu S Duh P Extraction and identification of antioxidant components from the leaves of mulberry (morus alba L.) Journal Agricultrual Food Chemistry 1996 44 1687 1690 10.1021/jf9503725

[B20] Ardestani A Yazdanparast R Antioxidant and free radical scavenging potential of achillea santolina extracts Food Chem 2007 104 21 29 10.1016/j.foodchem.2006.10.066

[B21] Djeridane A Yousfi M Nadjemi B Boutassouna D Stocker P Vidal N Antioxidant activity of some Algerian medicinal plants extracts containing Phenolic compounds Food Chem 2006 97 654 660 10.1016/j.foodchem.2005.04.028

[B22] Hussain AI Anwar F Shahid M Ashraf M Przybylski R Chemical composition, antioxidant and antimicrobial activities of essential oil of spearmint (mentha spicata L.) from Pakistan Journal Essential Oil Research 2010 22 78 84 10.1080/10412905.2010.9700269

[B23] Kordali S Cakir A Mavi A Kilic H Yildirim A Screening of chemical composition and antifungal and antioxidant activities of the essential oils from three Turkish Artemisia species J Agric Food Chem 2005 53 1408 1416 10.1021/jf048429n 15740015

